# The Role of Long Non-Coding RNAs in the Pathogenesis of Coronary Heart Disease

**DOI:** 10.3390/genes17070807

**Published:** 2026-07-15

**Authors:** Paulina Plewa, Joanna Kulpa, Jacek Szulc, Marcin Szczepanik, Maria Domańska, Andrzej Pawlik

**Affiliations:** Department of Physiology, Pomeranian Medical University, 70-111 Szczecin, Poland; paulina.plewa@op.pl (P.P.); joanna.h.kulpa@gmail.com (J.K.); marcin.t.szczepanik@gmail.com (M.S.); maria.domanska@onet.eu (M.D.)

**Keywords:** long non-coding RNA, coronary heart disease, MIAT, MALAT1, ANRIL, biomarker

## Abstract

Long non-coding RNAs (lncRNAs) constitute an important group of regulatory RNA molecules involved in the control of gene expression at the epigenetic, transcriptional, and post-transcriptional levels. In recent years, their crucial role in the pathophysiology of cardiovascular diseases, particularly coronary heart disease, has become increasingly evident. The aim of this review is to present current knowledge regarding the mechanisms of lncRNA action in the cardiovascular system, their involvement in the molecular processes associated with myocardial ischaemia, and their potential diagnostic and therapeutic applications. We discuss the molecular mechanisms responsible for the regulation of cardiomyocyte apoptosis, oxidative stress, mitochondrial dysfunction, angiogenesis, coronary vessel remodelling, and cardiac fibrosis. Particular attention is paid to selected lncRNAs involved in coronary heart disease, including MIAT, MALAT1, ANRIL, and H19, which influence inflammatory processes, vascular smooth muscle cell proliferation, responses to hypoxia, and cardiac fibrosis. The potential of lncRNAs as diagnostic and prognostic biomarkers in coronary artery disease is also discussed. Current evidence suggests that molecules such as MALAT1, MIAT, LIPCAR, and HCG11 may have considerable diagnostic and prognostic value, including for predicting major adverse cardiovascular events and the no-reflow phenomenon following percutaneous coronary intervention. Furthermore, contemporary therapeutic strategies targeting lncRNAs are presented, including antisense oligonucleotides, siRNAs, and CRISPR/Cas9 genome-editing technologies. Despite promising preclinical findings, the clinical application of lncRNA-based therapies remains limited by challenges related to safety, delivery of therapeutic molecules, and translation of experimental findings into clinical practice. Nevertheless, lncRNAs represent a promising avenue for the development of precision medicine and may play an important role in the future diagnosis and treatment of cardiovascular diseases.

## 1. Introduction

Coronary heart disease (CHD), also known as ischaemic heart disease (IHD), is the leading cause of disability and death worldwide. According to the latest analysis from the 2023 Global Burden of Disease study, cardiovascular deaths have increased significantly over the years. In 2023, cardiovascular disease was responsible for 19.2 million deaths, with IHD being the leading cause [1]. The increasing burden of IHD is largely attributable to population growth and ageing populations. Morbidity, mortality, and disability-adjusted life years were higher in men across all age groups, peaking in the oldest age group for both sexes [2,3]. The main factors contributing to IHD-related deaths and disability-adjusted life years were high systolic blood pressure, elevated glucose levels, high low-density lipoprotein (LDL) cholesterol levels, smoking, dietary factors, and environmental factors such as air pollution. The societal burden is greatest in countries with low and middle levels of sociodemographic development [1,3].

Atherosclerosis is the leading cause of CHD. Chronic inflammation, together with risk factors such as hypertension, oxidative stress, and hyperlipidaemia, increases the deposition of LDL, inducing immune cells to phagocytose and accumulate lipoproteins, ultimately forming foam cells [4]. Progressive changes in the vessel wall lead to the formation of atherosclerotic plaques with a lipid-necrotic core covered by a fibrous cap [5]. Atherosclerotic plaque growth can result in destabilisation and rupture, plaque erosion, or calcification. This initiates thrombus formation and vessel occlusion, leading to acute coronary syndromes [6].

Non-coding RNAs (ncRNAs) can be divided into short ncRNAs (<200 nucleotides) and long ncRNAs (lncRNAs) (>200 nucleotides), as shown in the table below. Together, they constitute nearly 80% of the genome (Table 1) [7,8,9,10].

ncRNAs play an important role in maintaining cardiovascular homeostasis by regulating gene expression at the transcriptional, translational, and post-transcriptional levels [7,11]. These molecules play fundamental roles in cardio-oncology, myocardial remodelling and fibrosis, angiogenesis, inflammation, and vascular injury, among other processes [11,12,13]. The growing understanding of their mechanisms of action highlights their importance and potential as targets for the development of therapies for cardiovascular diseases. The best-known ncRNAs involved in cardiac homeostasis are miRNAs, lncRNAs, and circRNAs. MicroRNAs regulate gene expression and are considered potential biomarkers of cardiovascular disease. Dysregulation of cellular pathways resulting from reduced miRNA expression significantly increases collagen production and fibrosis. In addition, miRNAs regulate genes involved in ion channel function, which is essential for proper cardiac electrical conduction and homeostasis [7]. circRNAs also influence myocardial contractility by regulating the expression of sarcoplasmic/endoplasmic reticulum Ca^2+^-ATPase (SERCA2a) and, consequently, calcium metabolism [14]. Their single-stranded, covalently closed structure confers exceptional stability [15]. Their functions are based primarily on acting as miRNA sponges and regulating splicing, transcription, and gene expression [16]. lncRNAs, which are the primary focus of this review, also play an important role in cardiac homeostasis. Their mechanisms of action are diverse and, therefore, their precise functions are not yet fully understood. They play a crucial role in chromatin regulation and gene expression through modulation of DNA–protein interactions. Similar to circRNAs, they also interact with miRNAs. In addition, they modulate mRNA splicing and stability and are therefore involved in cardiomyocyte regeneration, cardiac remodelling, apoptosis, and cardiomyocyte differentiation [11,17].

lncRNAs are non-coding transcripts exceeding 200 nucleotides in length that do not encode proteins. Their nomenclature includes molecules derived from processed introns and RNAs transcribed by RNA polymerases I, II, and III [18]. The current classification of lncRNAs is not entirely consistent because some lncRNAs perform multiple functions simultaneously and participate in several, often overlapping, molecular mechanisms. This complexity makes classifications based solely on molecular function, mechanism of action, subcellular localisation, transcriptional origin, or tissue specificity insufficient and potentially misleading. It is also worth noting that lncRNA classification is not standardised and continues to evolve [18,19,20]. Basic classification criteria include:

(A) Genomic location: lincRNAs (intergenic), intronic lncRNAs, enhancer-derived RNAs (eRNAs), and antisense lncRNAs (AS-lncRNAs) [21].
(B) Cellular location: Nuclear lncRNAs regulate transcriptional processes through chromatin organisation and participate in the formation of nuclear condensates by acting as scaffolds. Cytoplasmic lncRNAs modulate translation and regulate post-transcriptional gene expression. They influence mRNA stability and can sequester miRNAs [20,22,23].
(C) Mechanism of action: lncRNAs can act as:
  (1) Guides—by binding to proteins, other RNAs, and DNA and directing the resulting complex to a specific target. One example is the formation of a protein–DNA complex that modifies chromatin at a specific genomic location, leading to histone modification and transcriptional regulation [21,24,25].
  (2) Scaffolds—lncRNAs acting as scaffolds bind multiple proteins simultaneously, maintaining the stability of protein complexes. For example, MALAT1, interacts with phosphorylated serine/arginine-rich splicing factors in nucleus speckles, facilitating their recruitment to pre-mRNAs and ensuring constitutive splicing. Loss of MALAT1 results in accumulation of dephosphorylated SR proteins, shifting splicing towards alternative pre-mRNA splicing [16].
  (3) Decoys—lncRNAs that function as decoys bind and sequester regulatory proteins or RNAs. By binding regulatory molecules such as transcription factors, chromatin modifiers, or miRNAs, they prevent these molecules from reaching their target sites, thereby inhibiting their function [16,26].
  (4) miRNA sponges (ceRNAs)—competitive endogenous RNAs (ceRNAs), as their name suggests, function through the competitive binding of miRNA regulatory molecules. Because lncRNAs contain numerous miRNA-binding sites, they reduce miRNA availability for mRNA targets, thereby regulating gene expression [21,27].
(D) Range of regulation: cis-acting lncRNAs exert regulatory effects on nearby genes, typically through the regulation of chromatin structure or transcription factors. By contrast, trans-acting lncRNAs influence regulatory elements or genes located far from their site of origin [28].

## 2. Molecular Mechanisms in Myocardial Ischaemia

### 2.1. Apoptosis Regulation and Longevity of Cardiomyocytes

Morbidity and mortality associated with cardiovascular diseases are largely attributable to the limited regenerative capacity of cardiomyocytes [29,30]. Organismal homeostasis is maintained in part through apoptosis, one of the forms of programmed cell death (PCD). This process enables the elimination of unwanted, senescent, or dysfunctional cells, thereby maintaining tissue homeostasis and preventing cancer development. Unlike necrosis, apoptosis is an active process that requires energy expenditure [29,31]. PCD represents one of several pathways regulating cardiomyocyte death and contributes to the development of cardiovascular disease, although its precise role remains incompletely understood [29,32].

The PCD signalling pathway is as important for maintaining homeostasis as cell proliferation. Several forms of PCD have been identified, including apoptosis, necroptosis, autophagy, pyroptosis, and ferroptosis. Activation of different PCD pathways leads to distinct biological outcomes, although some pathways are interconnected [29,30]. Understanding how these pathways can be modulated may enable regulation of cardiomyocyte death and provide therapeutic benefits in the treatment of cardiovascular diseases. The unique electrophysiological and mechanical properties of cardiomyocytes are essential for maintaining cardiac function. Because cardiac muscle has little regenerative capacity, cardiomyocyte loss is largely irreversible, making cell death a major contributor to declining cardiac function and increased morbidity [29,30].

Apoptosis is the most common form of PCD under physiological conditions. It is characterised by cell shrinkage, increased cytoplasmic density, loss of mitochondrial membrane potential, and altered membrane permeability. Apoptosis can be initiated through two major pathways: the intrinsic apoptosis pathway (IAP), also known as the mitochondrial or Bcl-2-regulated pathway, and the extrinsic apoptosis pathway (EAP), also known as the death receptor pathway [29,33].

**Figure 1:**
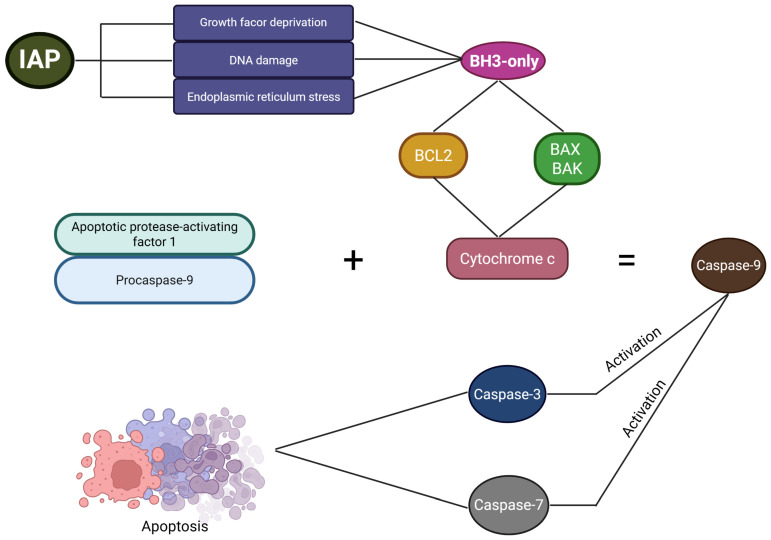
Schematic diagram of the intrinsic apoptosis pathway. The intrinsic apoptotic pathway is activated in response to cellular stress and leads to the activation of mechanisms responsible for programmed cell death. This process culminates in the activation of caspases and cell disintegration. Created in BioRender. Plewa, P. (2026) https://BioRender.com/atpqx5h. Accessed on 7 July 2026.

Triggers of the IAP include growth factor deprivation, DNA damage, and endoplasmic reticulum stress. These stimuli activate BH3-only proteins, which act as promoters of apoptosis and subsequently activate B-cell lymphoma 2 (Bcl-2) family proteins or the pro-apoptotic effectors BAX and BAK [29,34]. The Bcl-2 family comprises proteins that regulate apoptotic processes in both healthy and damaged cells and are localised primarily to the outer mitochondrial membrane [31,34]. Anti-apoptotic Bcl-2 proteins inhibit the activity of BAX and BAK. The tumour suppressor p53 can counteract this inhibition by activating BH3-only proteins [29,34]. p53 is involved in the activation of PCD, and oxidative stress increases its transcriptional activity, resulting in p53 accumulation. This promotes apoptosis by suppressing Bcl-2 expression and increasing BAX expression [29]. Under physiological conditions, p53 levels are controlled by the ubiquitin-proteasome system; however, cellular stress promotes translational and post-translational modifications that stabilise and activate p53 [34]. Following activation of Bcl-2 family proteins or the pro-apoptotic effectors BAX and BAK, mitochondria outer membrane permeabilization occurs, resulting in the release of cytochrome c [31]. Cytochrome c subsequently combines with apoptotic protease-activating factor 1 and procaspase-9 to form active caspase-9, which then activates caspases-3 and -7, ultimately initiating apoptosis (Figure 1) [29,31].

Caspases represent a key component in the regulation of PCD. These endoprotease enzymes mediate apoptotic signalling and execution [35,36]. Insufficient activation of caspases may impair host defence against infection, whereas excessive activation may contribute to inflammation and neurodegeneration. Caspases hydrolyse peptide bonds, generating active signalling molecules during apoptosis. Different caspases possess distinct protein interaction domains depending on whether they participate in the IAP or EAP. Caspases involved in the IAP contain a caspase recruitment domain, whereas those associated with the EAP possess a death effector domain [35]. In a study of rats with myocardial infarction (MI), caspases-3 and -9 were found to play important roles in cardiomyocyte apoptosis [33,36]. Through the activity of BAX, a key pro-apoptotic effector of the IAP, mitochondrial membrane permeability is increased. This accelerates apoptosis by promoting the release of pro-apoptotic proteins through membrane transition pores into the intermembrane space and subsequently into the cytosol. Apoptosis may then proceed through either caspase-dependent or caspase-independent mechanisms [34].

**Figure 2:**
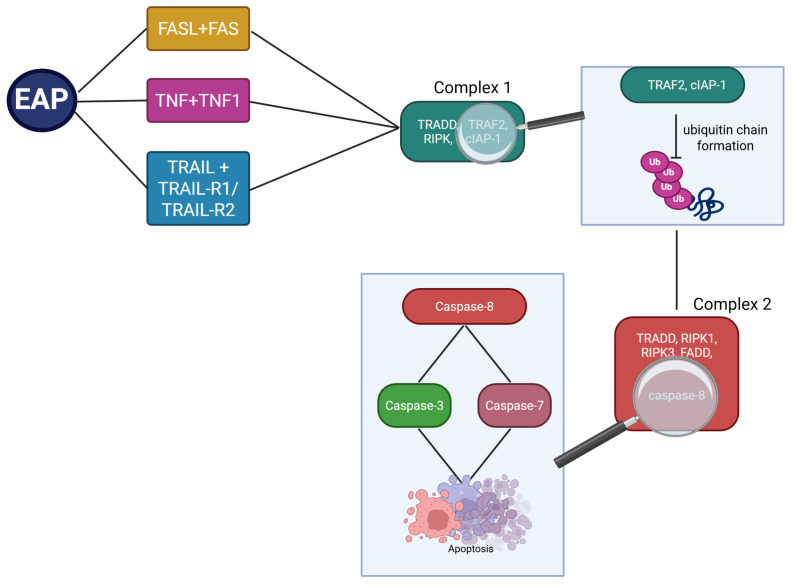
Schematic diagram of the extrinsic apoptosis pathway. The extrinsic apoptosis pathway begins after death ligands bind to membrane receptors, leading to the activation of caspase-8. Subsequently, executive caspases are activated, leading to cell apoptosis. Created in BioRender. Plewa, P. (2026) https://BioRender.com/6g4sxos. Accessed on 7 July 2026.

The EAP is initiated by the binding of death ligands, including FASL, TNF, and TRAIL, to their respective death receptors, FAS, TNFR1, TRAIL-R1, and TRAIL-R2. This interaction leads to the formation of complex I, composed of TRADD, TRAF2, RIPK1, and cIAP-1 [29]. Complex II is subsequently formed when TRAF2 and cIAP-1 inhibit ubiquitin chain formation. This complex consists of TRADD, RIPK1, RIPK3, FADD, and caspase-8 [29,33]. Caspase-8 activates caspases-3 and -7, thereby initiating apoptosis (Figure 2). It also activates BAX and BAK within the IAP and promotes the conversion of Bid to truncated Bid (tBid) [29]. By contrast, inhibition of caspase-8 leads to the activation of RIPK1, RIPK3, and MLKL. This process promotes the translocation of MLKL to the cell membrane, increasing membrane permeability and facilitating the release of damage-associated molecular patterns and pathogen-associated molecular patterns. Furthermore, MLKL activation can stimulate mitochondrial dynamin-related protein 1 via PGAM5, resulting in reactive oxygen species (ROS) accumulation and mitochondrial fission (Figure 2) [29].

In addition, lncRNAs participate in ischaemic remodelling during MI progression and influence biochemical pathways involved in cardiac function. In several studies during cardiac remodelling and during and after MI progression significant upregulation of myocardial infarct-associated transcript 1 and 2 (MIRT1 and MIRT2) was noted [37]. Furthermore, other lncRNA including aHIF, KCNQ1OT1, and MALAT1 were found to be elevated, similarly to MIRT1 and MIRT2, the conclusions drawn in both cases suggested a contribution of the lncRNA to the left ventricular remodelling process [37]. Certain lncRNAs have been proposed as biomarkers for MI, particularly cardiac apoptosis-related lncRNAs (CARLs), which inhibit mitochondrial fission, myocardial apoptosis in MI. CARLs are predominately localised in the cardiac tissue. After an MI CARLs exhibit elevated plasma levels compared to healthy individuals, making CARLS possible lncRNAs biomarkers for MI [37,38]. In MI, lncRNAs influence infarct size through the downregulation of solute carrier family 8 member A1 (SLC8A1), activation of the cGMP-PKG signalling pathway, and reduction in cytokine production [38]. LncRNAs play important regulatory roles at the epigenetic, transcriptional, and post-transcriptional levels by controlling genes involved in cardiomyocyte hypertrophy and contractility [37]. Additionally, lncRNAs modulate gene expression and cellular signalling through regulation of the phosphatidylinositol 3-kinase/protein kinase B (PI3K/AKT) pathway [38]. Furthermore, lncRNAs regulate gene expression through interactions with miRNAs and mRNAs, thereby functioning as ceRNAs [37,38]. One example is the interaction with miR-539, which leads to downregulation of PHB2 and suppression of MI-related injury [38].

### 2.2. Inflammatory Response in Cardiac Infarction

Reperfusion strategies are the standard treatment for MI, with the aim of restoring blood flow to ischaemic regions affected by vascular obstruction caused by atherosclerosis.

Nuclear factor kappa-light-chain-enhancer of activated B cells (NF-κB) comprises a family of transcription factors, including NF-κB1, NF-κB2, RelA, RelB, and c-Rel. NF-κB signalling operates through two major pathways: the canonical and non-canonical pathways [35,39]. NF-κB exists in both active and inactive forms, with the inactive form bound to inhibitory proteins [35]. In its inactive state, NF-κB is localised in the cytoplasm. Activation occurs in response to various stimuli, including oxidative stress. Following activation, the inhibitory proteins are phosphorylated and degraded, allowing NF-κB to translocate to the nucleus, where it regulates gene expression involved in inflammation and apoptosis [35]. NF-κB plays a key role in gene regulation, inflammatory responses, and cell proliferation. Its signalling pathway is also involved in cardiac regeneration through the regulation of cellular activity, myocardial fibrosis, and apoptosis. Suppression of NF-κB activation may therefore provide therapeutic benefits in several diseases. In cardiovascular disease and atherosclerosis, NF-κB is a major mediator of the inflammatory response, and targeting this pathway may help reduce cardiac fibrosis [35,40].

The NF-κB pathway can be activated by several stimuli, including ROS, hypoxia, and inflammatory cytokines. Once activated, it promotes further inflammatory responses through the induction of cytokines and recruitment of neutrophils, macrophages, T cells, and B cells, thereby contributing to cardiovascular tissue damage [35,39]. NF-κB is also involved in PCD through inhibition of pro-survival genes and induction of pro-death genes. Its activation promotes apoptosis by increasing the expression of inflammatory and apoptotic mediators, including C-reactive protein, interleukin (IL)-6, and tumour necrosis factor alpha (TNF-α) [29].

During the early stages following MI, macrophages predominantly exhibit a pro-inflammatory phenotype and promote cytokine production. As tissue repair progresses, a phenotypic shift occurs, and macrophages contribute to regenerative processes by supporting tissue restoration [41]. Macrophage activation begins following the infiltration of neutrophils into the damaged myocardium during acute MI, where macrophages participate in the clearance of cellular debris. Subsequently, changes in the local immune environment promote macrophage polarisation from the pro-inflammatory M1 phenotype to the reparative M2 phenotype. M2 macrophages release protective cytokines, including IL-2, IL-4, and IL-10, which contribute to repair and remodelling of the injured cardiac tissue [42].

### 2.3. Oxidative Stress and Mitochondrial Disfunction

Mitochondria are the primary energy-producing organelles of the cell and are particularly abundant in cardiac tissue because of its high energy demands. Recent studies have demonstrated that ROS and mitochondrial dysfunction play key roles in the pathophysiology of cardiovascular disease. Consequently, therapies targeting ROS production may represent an important strategy in the treatment of cardiac disorders. ROS include superoxide anions, hydroxyl radicals, and hydrogen peroxide. These molecules have dual roles in both physiological and pathological processes, and maintenance of intracellular redox homeostasis is essential for ensuring that ROS-mediated signalling activates only appropriate cellular pathways. Intracellular ROS levels are regulated by antioxidant defence systems, including superoxide dismutase, catalase, the glutathione peroxidase/reductase (GSH-PX) system, and the peroxiredoxin/thioredoxin (PRX/Trx) system [43,44].

ROS generation is associated with the activity of cytochrome P450 enzymes and occurs primarily in mitochondria and the endoplasmic reticulum. ROS are natural by-products of aerobic respiration and cellular oxygen metabolism. This family of reactive molecules includes superoxide anions, hydrogen peroxide, hydroxyl radicals, and nitric oxide. Excessive ROS production can promote inflammation, activate the NLRP3 inflammasome, and induce mitochondrial dysfunction [44,45]. ROS may arise from both exogenous and endogenous sources. Exogenous sources include radiation, smoking, and alcohol consumption, whereas mitochondria are the principal endogenous source through ATP synthesis during oxidative phosphorylation within the electron transport chain. The mitochondrial electron transport chain, located in the inner mitochondrial membrane, consists of five protein complexes: complex I, complex II, complex III, complex IV, and complex V. These are also known as NADH dehydrogenase (complex I), succinate dehydrogenase (complex II), ubiquinol-cytochrome c oxidoreductase (complex III), cytochrome c oxidase (complex IV), and ATP synthase (complex V), respectively. Electrons derived from NADH enter the electron transport chain through complex I, while complex II oxidises succinate to fumarate. Ubiquinone and cytochrome c function as electron carriers between the complexes. Ultimately, ATP synthase uses adenosine diphosphate (ADP) and inorganic phosphate to generate ATP in a process dependent on oxygen, which serves as the final electron acceptor in the electron transport chain. In addition to ATP production, superoxide anions and hydrogen peroxide are generated as by-products of oxidative phosphorylation. These ROS can induce oxidative damage to proteins and lipids, thereby impairing mitochondrial structure, function, and dynamics [44,45]. Oxidative stress resulting from excessive ROS accumulation can lead to genetic mutations and cellular damage. Consequently, ROS are considered important contributors to ageing and the development of cardiovascular diseases, as well as other disorders such as diabetes and neurodegenerative diseases [46].

### 2.4. Angiogenesis and Coronary Vessel Remodelling

Inflammation is a major risk factor for atherosclerosis. It initiates a cascade of events that begins with increased secretion of angiogenic factors, which bind to receptors on the endothelial surface, stimulating endothelial cell proliferation and migration [47]. Vascular endothelial growth factor (VEGF) is a family of signalling molecules involved in the regulation of blood and lymphatic vessel function. Owing to this role, VEGF contributes to the pathogenesis of atherosclerosis and related coronary heart diseases, including IHD and MI. The VEGF family comprises four major groups: VEGF-A, VEGF-B, VEGF-C, and VEGF-D, each with distinct biological functions. Regulation of angiogenesis is mediated by all four VEGF family members, although VEGF-A is considered the principal regulator of endothelial cell migration during this process. In addition to angiogenesis, VEGF is involved in vascular development, wound healing, tumour angiogenesis, and neovascularisation of atherosclerotic plaques [48]. VEGFA-LNC and VEGFC-LNC regulate the expression of VEGFA and VEGFC, respectively. Inhibition of VEGFA-LNC resulted in an approximately 1.8-fold increase in VEGFA expression. By contrast, deletion of VEGFC-LNC led to an approximately 1.6-fold reduction in VEGFC expression. Furthermore, VEGFC-LNC is predominantly localised within the nucleus. This lncRNA influences the expression of approximately 520 genes, either positively or negatively, although its mechanism of action does not appear to involve the 37 currently identified binding regions, suggesting the existence of additional indirect regulatory mechanisms [49]. These regulatory pathways may have therapeutic potential in coronary heart disease, particularly in compensating for hypoxia or ischaemia through controlled modulation of VEGF expression [50].

Hypoxia activates oxygen-sensing hypoxia-inducible factors (HIFs), among which hypoxia-inducible factor-1 alpha (HIF-1α) is particularly important. HIF-1α mediates vascular remodelling in response to hypoxia, making it a potential therapeutic target in ischaemic cardiovascular diseases. Both oxygen-dependent and oxygen-independent regulation of HIF-1α are critical for angiogenesis because HIF-1α promotes the expression of VEGF, glycolytic enzymes, and glucose transporters while reducing the expression of enzymes involved in the mitochondrial tricarboxylic acid cycle [51]. The effects of HIF-1α depend on both the magnitude and duration of its activation. Transient activation promotes adaptive responses, including angiogenesis, whereas sustained or dysregulated activation may contribute to maladaptive remodelling, fibrosis, and arrhythmogenesis [52]. Several lncRNAs directly regulate HIF-1α signalling. For example, GATA2-AS1 regulates the balance between active HIF-1α and HIF-2α, thereby influencing endothelial responses to both acute and chronic stress. GATA2-AS1 shifts this balance in favour of HIF-1α, promoting activation of glycolytic pathways and modulation of mitochondrial biogenesis. In atherosclerosis, however, these GATA2-AS1-mediated regulatory pathways appear to be impaired [53]. Another lncRNA with an important role under hypoxic conditions is H19. Expression of H19 is induced by HIF-1α, which is itself elevated during cellular stress. Interestingly, these molecules exhibit a reciprocal relationship, with H19 stabilising HIF-1α. Experimental blockade of H19 has been shown to attenuate cellular injury [54]. HIF1A-AS is located near the HIF1A locus and is strongly upregulated during hypoxia. Unlike H19, it acts antagonistically to HIF-1α by suppressing its transcriptional activity [55].

### 2.5. Remodelling and Fibrosis of the Cardiac Muscle

Structural changes induced by acute or chronic diseases that affect the integrity of the heart muscle are a major contributor to the high mortality associated with CHD. Therefore, regulation of cardiac fibrosis is an important therapeutic target for improving clinical outcomes [56,57,58].

Cardiac fibrosis is characterised by excessive deposition of extracellular matrix proteins, predominantly collagen types I and III, which impairs cardiac function [38,56,57]. Cardiac fibrosis can be classified into two main forms: reparative and interstitial [56,57]. Reparative cardiac fibrosis occurs following ischaemic injury and results in the formation of scar tissue that replaces oxygen-deprived myocardial tissue lost through necrosis and apoptosis. Reparative cardiac fibrosis is an essential repair process because it preserves the structural integrity of the heart by replacing damaged tissue and limiting further injury [57]. By contrast, interstitial cardiac fibrosis is typically associated with chronic disease and contributes to alterations in cardiac function, including impaired ventricular compliance, diastolic function, and disrupted electrical connectivity [56].

Cardiac remodelling refers to structural and functional alterations that occur in response to pathological stimuli affecting the heart. It is particularly evident following MI, where coronary artery occlusion leads to myocardial cell death and subsequent necrosis. In response, surviving cardiomyocytes undergo hypertrophic growth, while necrotic tissue is progressively replaced by extracellular matrix components [38,59]. Cardiac fibroblasts play a central role in the healing and remodelling process after MI. However, fibrotic tissue lacks the contractile properties and electrical conductivity of normal myocardium. Consequently, the remodelled region cannot effectively conduct electrical impulses, increasing the risk of arrhythmias and contributing to sudden cardiac death [57,59]. Over time, continued myocardial remodelling and fibrosis lead to thickening of the cardiac wall and increased ventricular pressure overload as a compensatory response to reduced contractile function. Preservation of cardiac function may therefore be achieved through inhibition of myocardial fibrosis. Excessive myocardial fibrosis progressively impairs cardiac performance and accelerates the transition to decompensated heart failure [57,59]. Cardiac fibroblasts are responsible for extracellular matrix turnover and account for approximately 20% of all cardiac cells [59,60]. lncRNAs have emerged as important regulators of cardiac remodelling and fibrosis. For example, the lncRNA H19 promotes fibrosis by sequestering let-7 microRNAs and enhancing transforming growth factor beta 1 (TGF-β1) signalling, thereby accelerating fibrotic processes within the myocardium [38].

## 3. Selected lncRNAs Involved in CHD

### 3.1. MI-Associated Transcript (MIAT)

MIAT was first identified in 2006 as a lncRNA that plays an important role in the development of MI [61]. Since then, numerous studies have demonstrated upregulation of MIAT in coronary artery disease (CAD) [62,63,64]. MIAT is involved in multiple aspects of CAD pathophysiology.

**Figure 3:**
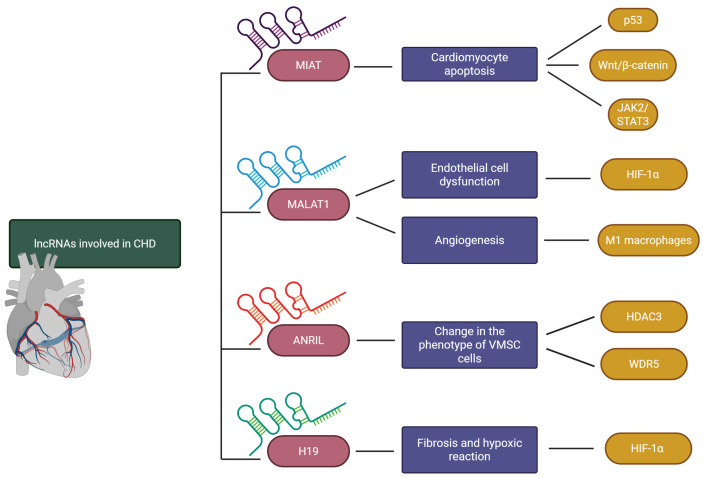
The role of selected lncRNAs in the pathogenesis of CHD. MIAT influences cardiomyocyte apoptosis, MALAT1 participates in endothelial cell dysfunction and angiogenesis, ANRIL is responsible for changing the VSMC phenotype, and H19 participates in fibrosis and the response to hypoxia. Created in BioRender. Plewa, P. (2026) https://www.biorender.com/. Accessed on 29 May 2026.

Cardiomyocyte apoptosis is a process of major importance in the pathophysiology of CAD [64] and has been shown to be regulated by MIAT. Hayasaka et al. [64] demonstrated that the expression of the apoptotic markers p53, Bak1, and cleaved caspase-3 was reduced in cardiomyocyte-specific MIAT knockout mice following MI compared with control animals. Hypoxia is one of the principal factors promoting cardiomyocyte apoptosis in CAD, and Zhang et al. [65] investigated the regulatory role of MIAT in this process. Their study demonstrated that exposure of cardiomyocytes to hypoxia upregulates MIAT, which binds to miR-488-3p and prevents its interaction with Wnt5a mRNA. This allows Wnt5a translation and activation of the canonical Wnt/β-catenin signalling pathway [65]. In the canonical Wnt pathway, binding of a Wnt ligand to its receptor complex prevents β-catenin phosphorylation, resulting in β-catenin translocation to the nucleus, where it regulates transcription of target genes [66]. Activation of the Wnt/β-catenin pathway has been shown to induce cardiomyocyte apoptosis under hypoxic conditions [65]. Shen et al. [67] investigated the effects of MIAT on p53 signalling in hypoxia-exposed cardiomyocytes. p53 is a critical regulator of PCD [67]. It promotes the IAP, which is activated by DNA damage, growth factor deprivation, and endoplasmic reticulum stress. p53 exerts its effects primarily through upregulation of BH3-only proteins of the Bcl-2 family, which inhibit pro-survival proteins [68]. The study by Shen et al. [67] demonstrated that hypoxia-induced upregulation of MIAT in cardiomyocytes results in binding of MIAT to miR-708-5p, thereby preventing miR-708-5p from interacting with p53 mRNA. This promotes p53 expression, leading to increased cardiomyocyte apoptosis [67]. Another pro-apoptotic mechanism was investigated in a study by Tan et al. [69], which demonstrated that under oxygen-glucose deprivation conditions MIAT promotes cardiomyocyte apoptosis by inhibiting miR-181a-5p and consequently activating the Janus kinase 2 (JAK2)/signal transducer and activator of transcription 3 (STAT3) signalling pathway. Interestingly, JAK2/STAT3 signalling has also been reported to reduce cardiomyocyte apoptosis in cardiovascular diseases, suggesting that its effects depend on the broader cellular and molecular context (Figure 3) [70].

Endothelial cell dysfunction is another important component of CAD pathophysiology that is regulated by MIAT. In a study by Li et al. [71], MIAT expression in endothelial cells was shown to be induced by oxidised LDL (oxLDL). Through inhibition of miR-214-3p, MIAT increased expression of caspase-1, a pro-inflammatory caspase that plays a central role in pyroptosis, a form of inflammatory programmed cell death [71]. Depending on the intracellular oxidative environment, caspase-1 may also promote apoptosis [72]. Li et al. [71] further demonstrated that oxLDL-induced upregulation of MIAT enhanced endothelial cell apoptosis and increased expression of IL-1β.

### 3.2. Metastasis-Associated Lung Adenocarcinoma Transcript 1 (MALAT1)

As discussed above, endothelial cell dysfunction plays a vital role in the development of CAD and has been shown to be regulated by MALAT1. Liu et al. [73] demonstrated in human umbilical vein endothelial cells that under hypoxic conditions, MALAT1 promotes apoptosis, autophagy, and the expression of pro-inflammatory cytokines. These effects are mediated through inhibition of miR-19b-3p, resulting in upregulation of HIF-1α [73]. HIF-1α is a subunit of HIF-1, a key regulator of cellular responses to oxidative stress that has been implicated in the promotion of endothelial barrier dysfunction under hypoxic conditions (Figure 3) [74].

MI is one of the acute manifestations of CAD and triggers a cascade of pathophysiological changes involving multiple cell types, collectively referred to as post-MI ventricular remodelling. This process comprises an early phase characterised by extracellular matrix degradation and a later phase associated with cardiomyocyte hypertrophy and collagen scar formation [75]. MALAT1 has been shown to increase both the extent of fibrosis and the expression of pro-inflammatory cytokines in mice following MI [76,77]. In addition, MALAT1 influences post-MI angiogenesis, which is an important component of ventricular remodelling [78,79], as MI causes substantial damage to the cardiac microcirculation. Macrophages and fibroblasts are among the principal cell types involved in regulating post-MI angiogenesis [80]. Pro-inflammatory M1 macrophages may modulate post-MI remodelling through the release of extracellular vesicles (EVs) (Figure 3). Chen et al. [78] demonstrated in a murine MI model that EVs derived from M1 macrophages exacerbate post-infarction cardiac dysfunction, fibrosis, and aberrant angiogenesis. Investigation of the underlying mechanism in oxygen-glucose-deprived microvascular endothelial cells (MMECs) revealed a regulatory role for MALAT1. M1 macrophages secrete EVs containing MALAT1, which inhibits miR-25-3p in MMECs, thereby increasing expression of the target gene encoding cell division control protein 42 (CDC42) and aggravating post-MI remodelling [78]. The role of MALAT1 in post-MI remodelling remains complex, however; evidence also suggests protective effects. Chen et al. [79] demonstrated that MALAT1 facilitates post-MI tissue repair through modulation of microvascular function. MALAT1-knockdown mice exhibited lower survival rates and reduced microvascular perfusion compared with control animals. Furthermore, the beneficial effects of MALAT1 on endothelial cells after MI were shown to be mediated, at least in part, through preservation of mitochondrial function via the MALAT1/miR-26b-5p/mitofusin 1 axis [79]. Mitofusin 1 is a tethering protein that regulates mitochondrial dynamics through its role in mitochondrial fusion. It is also important for maintaining endothelial cell viability and angiogenic capacity [80]. By upregulating mitofusin 1, MALAT1 reduced mitochondrial fragmentation, decreased mitochondrial debris accumulation, and lowered the expression of mitochondria-associated apoptotic genes in cardiac microvascular endothelial cells [79].

### 3.3. Antisense ncRNA in the INK4 Locus (ANRIL)

ANRIL is encoded within the 9p21.3 locus, a genomic region strongly associated with cardiovascular diseases. The 9p21.3 risk haplotype in vascular smooth muscle cells (VSMCs) has been linked to pro-atherogenic cellular characteristics [81]. ANRIL regulates the pathophysiological functions of VSMCs in CAD through several mechanisms.

VSMCs exist in two principal phenotypes: contractile and synthetic. The contractile phenotype predominates under physiological conditions and is characterised by low proliferative activity, whereas the synthetic phenotype is associated with pathological conditions such as CAD and exhibits increased proliferation and protein synthesis [82,83]. Zhang et al. [84] demonstrated that ANRIL promotes oxLDL-induced phenotypic switching in human aortic smooth muscle cells (HASMCs). Compared with cells expressing normal levels of ANRIL, ANRIL-knockdown HASMCs exhibited reduced proliferation, migratory activity, ROS production, and expression of synthetic phenotype markers. The authors also identified a potential molecular mechanism underlying these effects. ANRIL acts as a scaffold by recruiting WD repeat-containing protein 5 (WDR5) and histone deacetylase 3 (HDAC3) to the promoter region of NADPH oxidase 1 (NOX1), thereby facilitating gene transcription. This results in increased NOX1 expression, which promotes ROS production and induces VSMC phenotypic switching [84]. Both WDR5 and HDAC3 are known regulators of gene expression through their effects on chromatin structure (Figure 3) [85]. In addition, WDR5 functions as a methyltransferase-associated protein that enhances the activity of H3K4 methyltransferases [85]. Huang et al. demonstrated that ANRIL can also promote the proliferation of oxLDL-treated HASMCs by acting as a molecular sponge for miR-339-5p. Sequestration of this miRNA results in upregulation of fibroblast growth factor receptor substrate 2 (FRS2), a scaffold adaptor protein involved in activation of the proliferation-associated RAS/RAF/ERK signalling pathway [86,87].

### 3.4. H19

H19 is involved in CAD through its roles in fibrosis, cardiac hypertrophy, and cellular responses to hypoxia [54,88,89]. Xie et al. demonstrated that H19 interacts with HIF-1α and influences cardiomyocyte function under hypoxic conditions. As discussed previously, HIF-1α is a subunit of HIF-1, a key regulator of cellular adaptation to hypoxia. Under hypoxic conditions, HIF-1α is stabilised and binds to constitutively expressed HIF-1β to form the HIF-1 complex, which subsequently translocates to the nucleus and regulates target gene expression through binding to hypoxia response elements within promoter regions [54,90]. The study demonstrated that hypoxia-induced HIF-1α promotes transcription of H19 in cardiomyocytes. Furthermore, H19 contributes to stabilisation of HIF-1α under hypoxic conditions by preventing its proteasomal degradation. Consequently, the interaction between HIF-1α and H19 constitutes a positive feedback loop. This reciprocal regulation promotes cardiomyocyte death, mitochondrial dysfunction, and ROS production during hypoxia (Figure 3) [54].

CAD and the associated process of cardiac remodelling are frequently accompanied by cardiac hypertrophy. Although hypertrophy initially serves as a compensatory mechanism, excessive and sustained hypertrophic growth ultimately contributes to cardiac dysfunction. Cardiomyocyte hypertrophy is also a key feature of late-stage post-MI remodelling [75,91,92]. In a study by Viereck et al., H19 was shown to exert protective effects against cardiac hypertrophy both in vitro and in vivo. Mechanistically, H19 inhibits methylation of the tescalcin promoter through suppression of enhancer of zeste homologue 2 (EZH2), a component of polycomb repressive complex 2 (PRC2) responsible for establishing repressive histone modifications. During cardiac remodelling, H19 expression is reduced, resulting in increased tescalcin promoter methylation and transcriptional repression. Loss of tescalcin, a negative regulator of pro-hypertrophic genes, consequently promotes cardiac hypertrophy [88,93].

Fibrosis is another important component of CAD pathophysiology and a hallmark of late-stage post-MI remodelling [75]. H19 has been shown to exert protective effects against cardiac fibrosis in a rat model of MI. Overexpression of H19 reduced infarct size, attenuated post-MI fibrosis, and improved cardiac function compared with control animals. Mechanistically, H19 targets miR-22-3p, resulting in upregulation of lysine-specific demethylase 3A (KDM3A), a histone-modifying enzyme that demethylates mono-methylated and di-methylated histone H3 lysine 9 (H3K9) residues [89,94].

### 3.5. Associated circRNAs

Circular RNAs are a separate group of non-coding RNAs which may affect CAD by interacting with the same miRNAs as lncRNAs. Zeng et al. demonstrated that in human coronary artery SMCs circular RNA MAP3K5 (circMAP3K5) targets miR-22-3p, which is also known to be targeted by H19. Sequestration of miR-22-3p by circMAP3K5 prevents miR-22-3p from inhibiting Tet methylcytosine dioxygenase 2 (TET2). TET2 is a DNA modifying enzyme oxidising 5-methylcytosine (5-mC) to 5-hydroxymethylcytosine (5-hmC) which allows for demethylation and activation of genes associated with contractile phenotype of SMCs [89,95,96].

As mentioned above MIAT targets miR-214-3p in endothelial cells. This miRNA was also demonstrated in HUVECs hypoxia-reoxygenation model to be sponged by circular RNA ZNF609 (circZNF609). Hypoxia-reoxygenation injury in vitro model is used to investigate injuries caused by reperfusion after a period of ischemia in myocardial infarction. Sponging of miR-214-3p results in upregulation of a well-known inflammation-associated enzyme cyclooxygenase 2 (COX-2), which aggravates the hypoxia-reoxygenation injury. Moreover, knockdown of circZNF609 in a murine model reduced myocardial ischemia reperfusion injury [97]. Hsa_circ_0029589 is another circRNA targeting miR-214-3p in coronary artery disease. Sequestering of miR-214-3p by this circRNA upregulates stromal interaction molecule 1 (STIM1), thereby promoting VSMCs proliferation and migration, which are characteristic features of atherosclerotic synthetic phenotype [98]. In addition, a study by Cui et al. showed that effect of STIM1 on VSMCs proliferation and migration may be exerted through upregulation of a calcium channel called Orai1 [99]. Circular RNA CFHR was demonstrated to also sponge miR-214-3p in VSMCs. In a study by Zhuang et al. sequestering of this miRNA by circCFHR allowed for activation of Wnt3/β-Catenin pathway and promotion of VSMCs growth, migration and inflammatory responses [100].

## 4. lncRNAs as Biomarkers in CHD

Numerous lncRNAs are upregulated in the blood of patients with CAD, and a growing body of evidence suggests that they may serve as diagnostic biomarkers for cardiovascular diseases, including CAD. Examples include MIAT, MALAT1, ANRIL LINC00963, and SNHG15 [101,102,103]. In a meta-analysis comprising 10 studies, MALAT1 and MIAT demonstrated moderate diagnostic value for CAD, with pooled areas under the curve (AUCs) of 0.746 and 0.757, respectively [101]. In a study by Saberiyan et al. [102], long intergenic non-protein coding RNA 963 (LINC00963) was reported to be an excellent diagnostic biomarker for CAD, with an AUC of 0.9424. By contrast, long intergenic non-protein coding RNA 1220 (LINC01220) was found to be downregulated in the blood of patients with CAD and showed fair diagnostic performance for early CAD, with an AUC of 0.796 (95% CI: 0.718–0.874; sensitivity 51.6%; specificity 91.7%) [104]. In addition, plasma exosomal lncRNAs may also have diagnostic utility. For example, circulating exosomal lncRNA ENST00000560769.1 demonstrated fair diagnostic efficacy, with an AUC of 0.722 ± 0.048 (95% CI: 0.627–0.817, *p* < 0.001) [105].

Several lncRNAs have also shown promise as biomarkers for monitoring disease progression and assessing prognosis following MI. Long intergenic non-coding RNA predicting cardiac remodelling (LIPCAR) and MALAT1 demonstrated considerable prognostic accuracy for predicting major adverse cardiovascular events after ST-segment elevation MI (STEMI), with AUCs of 0.815 and 0.792, respectively. Combined assessment of these biomarkers further improved prognostic performance (AUC = 0.842) [106].

Percutaneous coronary intervention (PCI) is the standard treatment for STEMI. However, in some patients, adequate myocardial perfusion is not restored despite successful reopening of the occluded artery, a phenomenon known as no-reflow. Yang et al. [107] demonstrated that MALAT1 could serve as a biomarker for predicting no-reflow after PCI, with an AUC of 0.95. Therefore, MALAT1 may potentially aid in identifying patients at increased risk of no-reflow following PCI [107]. HCG11 is another lncRNA with potential prognostic value in patients with STEMI treated by PCI. HCG11 demonstrated promising predictive performance for major adverse cardiovascular events after PCI-treated MI, with an AUC of 0.896 and sensitivity and specificity values of 81.6% and 88.7%, respectively. When combined with miR-532-3p, its predictive value increased further, achieving an AUC of 0.958, with sensitivity and specificity of 84.2% and 95.2%, respectively [108].

Interestingly, not only long non-coding ANRIL, but also circular ANRIL (circANRIL) is a promising biomarker. In contrast with lncANRIL circANRIL is downregulated in CAD patients [109]. In a study by Fang et al., circANRIL(exon14-4) was demonstrated to have a fair diagnostic value for CAD with an AUC of 0.713 (95% CI 0.644–0.781, *p* < 0.001), and sensitivity, and specificity of 94.6% and 35.9%, respectively. Moreover, lower expression of circANRIL(exon14-4) was an independent risk factor of major adverse cardiovascular events (HR 0.11, 95% CI 0.02–0.54, *p* < 0.01) [110].

In summary, lncRNAs show considerable promise as both diagnostic and prognostic biomarkers in IHD. Further studies focusing on lncRNAs with the highest diagnostic and prognostic performance are warranted to facilitate their translation into clinical practice.

## 5. Therapeutic Potential of Targeting lncRNAs

### 5.1. Antisense Oligonucleotides (ASOs)

ASOs are single-stranded nucleic acid polymers that can be broadly classified into two categories: RNase H-competent ASOs and steric-blocking ASOs. Both approaches rely on binding to target RNA molecules, but they exert their effects through distinct mechanisms. RNase H-competent ASOs recruit the endogenous RNase H1 (RNASEH1) enzyme, which cleaves the RNA strand within the RNA-DNA duplex, leading to degradation of the target transcript. In contrast, steric-blocking ASOs bind to pre-mRNA and modulate splicing by preventing access of the splicing machinery to specific sites, thereby promoting exon inclusion or exon skipping [111].

One potential therapeutic target for ASO-based therapy is WISPER, a lncRNA expressed predominantly in cardiac fibroblasts. WISPER has been shown to regulate cardiac fibrosis following myocardial injury. Its involvement in fibrotic remodelling has been demonstrated in both murine models of MI and human cardiac biopsy samples, where WISPER expression correlated with the degree of fibrosis. In vivo administration of ASOs targeting WISPER reduced both cardiac dysfunction and fibrosis after MI. Mechanistically, WISPER regulates the expression of lysyl hydroxylase 2, a profibrotic factor involved in extracellular matrix remodelling within the heart [112].

ASO technology may also be applicable to MIAT, a lncRNA that modulates the cardioprotective microRNA miR-150. Knockdown of MIAT has been shown to attenuate the development of MI and adverse cardiac remodelling. Moreover, miR-150 acts antagonistically to MIAT by suppressing excessive maladaptive remodelling. One of its targets is HOXA4, whose expression is promoted by MIAT. HOXA4 exerts profibrotic effects, and its inhibition may improve cardiac function and reduce pathological remodelling [113].

### 5.2. siRNA-Based Therapies

siRNA is a double-stranded RNA molecule typically consisting of approximately 21 base pairs and composed of a sense strand and a guide strand. Following cellular uptake through endocytosis, siRNA associates with the RNA-induced silencing complex (RISC). Within this complex, a member of the argonaute (AGO) protein family, primarily AGO2, recognises the guide strand and promotes degradation of the complementary sense strand. AGO2 contains three major functional domains: PAZ, MID, and PIWI. The PAZ and MID domains recognise the 3′ and 5′ ends of the guide strand, respectively, whereas the PIWI domain possesses endonuclease activity and mediates cleavage of the target mRNA [114].

Cardiac hypertrophy is regulated in part by the lncRNA cardiac hypertrophy-related factor (CHRF; AK048451), which is located within the first intron of the Dcc (deleted in colorectal carcinoma) gene in mice. CHRF directly binds miR-489 and regulates its activity. Elevated CHRF expression has been observed in cardiac biopsy specimens and animal models of heart failure, including models of transverse aortic constriction, as well as following angiotensin II administration. Silencing CHRF using siRNA increases miR-489 expression and activity. In addition, suppression of CHRF reduces expression of myeloid differentiation primary response protein 88 (MyD88), linking this lncRNA to inflammatory signalling and apoptotic pathways. These findings suggest that silencing CHRF may be a potential therapeutic strategy for reducing disease severity [115].

Downregulation of another lncRNA, ANRIL, reduced the number of structurally abnormal cardiomyocytes. Silencing ANRIL may also attenuate cardiac fibrosis and remodelling. Furthermore, ANRIL silencing increased expression of the anti-apoptotic protein Bcl-2 while reducing the levels of the pro-apoptotic proteins caspase-3 and Bax, suggesting a reduction in cardiomyocyte apoptosis following siRNA treatment [116]. These findings may be very important factors in prevention of CHD.

### 5.3. CRISPR/Cas9

CRISPR/Cas9 technology is a promising approach for addressing both the severity and underlying causes of CHD. Clustered regularly interspaced short palindromic repeats (CRISPR) is a novel genome-editing technology. The system consists primarily of two components: the Cas9 endonuclease, which introduces double-stranded DNA breaks, and a guide RNA, a fragment of approximately 100 nucleotides in length. The guide RNA directs the Cas9 enzyme to a complementary DNA sequence, where Cas9 generates a double-strand break. In response, the cell activates one of two repair mechanisms. The first, non-homologous end joining, ligates the DNA ends without the use of a repair template and often results in random nucleotide insertions or deletions, potentially causing frameshift mutations. The second mechanism, homology-directed repair, uses an exogenous repair template and allows more precise genetic modifications [117,118,119].

Cardiac hypertrophy–associated epigenetic regulator (Chaer) has been proposed as a potential target for CRISPR/Cas9-mediated silencing. Chaer is an essential regulator of cardiac hypertrophy and directly modulates PRC2, thereby influencing methylation of genes associated with hypertrophic growth. Experimental studies have shown that downregulation of Chaer before the onset of pathological stress reduces cardiac hypertrophy and dysfunction [120]. These findings suggest that Chaer may be a suitable candidate for therapeutic genome editing using CRISPR/Cas9 technology.

Another lncRNA for which inhibition may provide therapeutic benefit is FOXF1 adjacent non-coding developmental regulatory RNA (Fendrr). Although Fendrr has physiological functions in healthy murine hearts and exhibits anti-fibrotic properties in pulmonary fibrosis, its role appears to differ in cardiac fibrosis. In mouse models of cardiac fibrosis induced by transverse aortic constriction, Fendrr expression was increased. Loss-of-function studies demonstrated attenuation of the fibrotic response. Mechanistically, Fendrr acts through the miR-106b/Samd3 signalling pathway, in which these molecules exert opposing effects. Overexpression of miR-106b has been shown to alleviate cardiac fibrosis and reduce the abnormal expression of collagen types I and III [121].

## 6. Discussion

lncRNAs play a significant role in the regulation of cardiovascular homeostasis and in the pathogenesis of CAD. Numerous studies have demonstrated their involvement in key biological processes, including cardiomyocyte apoptosis, inflammatory responses, oxidative stress, mitochondrial dysfunction, angiogenesis, fibrosis, and myocardial remodelling. Of particular importance are lncRNAs such as MIAT, MALAT1, ANRIL, and H19, which regulate gene expression through interactions with miRNAs, regulatory proteins, and chromatin-associated factors. Growing evidence also suggests that lncRNAs may serve as valuable diagnostic and prognostic biomarkers in CAD.

Therapeutic strategies targeting lncRNAs represent another important area of ongoing research. ASOs, siRNA-based approaches, and CRISPR/Cas9 genome-editing technologies have shown promising results in preclinical models by modulating the expression of lncRNAs involved in fibrosis, cardiac remodelling, and cardiomyocyte apoptosis. Current findings suggest that selective targeting of disease-associated lncRNAs may contribute to the development of more personalised therapies for cardiovascular diseases in the future.

Despite substantial progress, several limitations continue to hinder the clinical translation of lncRNA-based therapies. One of the major challenges is the efficient and safe delivery of therapeutic molecules to the myocardium. Lipid nanoparticles often exhibit limited tissue specificity, and some therapeutic approaches require direct myocardial administration. In addition, certain delivery systems may provoke immune responses, including activation of Toll-like receptors or reactions associated with polyethylene glycol. Off-target effects and non-specific distribution of RNA-based therapeutics to other organs and immune cell populations also remain significant obstacles that must be addressed before widespread clinical application can be achieved.

Another limitation is the difficulty of translating preclinical findings into clinical practice. A substantial proportion of lncRNAs exhibit species-specific expression and are absent in commonly used laboratory models such as mice and rats, making it challenging to evaluate their true biological functions in humans. Accurate modelling of the pharmacokinetics and pharmacodynamics of ASOs and siRNA-based therapies also remains problematic. Although physiologically based pharmacokinetic and pharmacokinetic/pharmacodynamic models have shown promising predictive value, further refinement and validation are still required.

In the future, multicentre phase I–III clinical trials will be essential for evaluating the safety, efficacy, and long-term effects of lncRNA-based therapies. Further advances in understanding the molecular mechanisms regulated by lncRNAs, together with the development of more selective and stable delivery systems, will also be necessary. Progress in sequencing technologies, molecular biology, and genome-editing tools may facilitate the development of novel targeted therapies and support the integration of lncRNA-based approaches into the future management of cardiovascular diseases.

## Figures and Tables

**Table 1:** RNA division.

Group	RNA Type	Function and Characteristics	References
Constitutive RNA	Transfer RNA (tRNA)	Transports amino acids to ribosomes	[7,8,9,10,11,12,13,14,15,16,17]
	Ribosomal RNA (rRNA)	Main component of ribosomes; catalyses peptide bond formation	
	Small nuclear RNA (snRNA)	Participates in intron excision	
Short regulatory (<200 nt)	MicroRNA (miRNA)	Regulates gene expression at the post-transcriptional level	
	Small interfering RNA (siRNA)	Silences genes by RNA interference (RNAi)	
	Small nucleolar RNA (snoRNA)	Directs chemical modifications of rRNA/mRNA	
	Piwi-interacting RNAs (piRNAs)	Regulates gene expression in somatic tissues	
Long regulatory (>200 nt)	Long non-coding RNAs (lncRNAs)	Regulates chromatin structure, transcription, and RNA availability	
	Circular RNA (circRNA)	miRNA sequestration, modulation of splicing, and interaction with RNA-binding proteins (RBPs)	

## Data Availability

No new data were created or analyzed in this study. Data sharing is not applicable to this article.

## Abbreviations

The following abbreviations are used in this manuscript:

AUC	Areas under the curve
ANRIL	Antisense ncRNA in the INK4 locus
Bcl-2	B-cell lymphoma 2
CAD	Coronary artery disease
CDC42	Cell division control protein 42
CHD	Coronary heart disease
EAP	Extrinsic apoptosis pathway
EVs	Extracellular vesicles
EZH	Enhancer of zeste homologue
FRS	Fibroblast growth factor receptor substrate
GSH-PX	Glutathione peroxidase/reductase
HASMCs	Human aortic smooth muscle cells
HDAC	Histone deacetylase
HIFs	Hypoxia-inducible factors
IAP	Intrinsic apoptosis pathway
IHD	Ischaemic heart disease
IL	Interleukin
JAK	Janus kinase
LDL	Low-density lipoprotein
LINC01220	Long intergenic non-protein coding RNA 1220
lncRNA	Long non-coding RNA
MALAT1	Metastasis-associated lung adenocarcinoma transcript 1
MI	Myocardial infarction
MIAT	MI-associated transcript
MMECs	Muscle microvascular endothelial cells
ncRNA	Non-coding RNA
NF-κB	Nuclear factor kappa-light-chain-enhancer of activated B cells
oxLDL	Oxidised LDL
PCD	Programmed cell death
PCI	Percutaneous coronary intervention
PI3K/AKT	Phosphatidylinositol 3-kinase/protein kinase B
PRC	Polycomb repressive complex
PRX/Trx	Peroxiredoxin/thioredoxin
RISC	RNA-induced silencing complex
ROS	Reactive oxygen species
SERCA2a	Sarcoplasmic/endoplasmic reticulum Ca2+-ATPase
SLC8A1	Solute carrier family 8 member A1
STAT	Signal transducer and activator of transcription
STEMI	ST-segment elevation MI
TNF-α	Tumour necrosis factor alpha
VEGF	Vascular endothelial growth factor
VSMCs	Vascular smooth muscle cells
